# Cholera Infantum

**Published:** 1879-04

**Authors:** 


Cholera Infantum.—During the summer of 1873, I was called to prescribe for a child two years old, supposed by the physician in attendance to be dying, the disease being diagnosed as cholera infantum. My prescription was one ripe strawberry every hour till better. The child speedily recovered. Three months after, I was asked to prescribe for another child aged eleven months. The disease this time was really cholera infantum. One-half strawberry every hour proved a successful treatment. This child had also been given up to die.—*Boston Journal of Chemistry.*

